# Bullying in Primary School Children: The Relationship between Victimization and Perception of Being a Victim

**DOI:** 10.3390/ijerph17249540

**Published:** 2020-12-20

**Authors:** Francesc Sidera, Elisabet Serrat, Jordi Collell, Georgina Perpiñà, Robinson Ortiz, Carles Rostan

**Affiliations:** 1 Psychology Department, University of Girona, 17004 Girona, Spain; elisabet.serrat@udg.edu (E.S.); georgina.perpinya@udg.edu (G.P.); hrobinsonc@gmail.com (R.O.); carles.rostan@udg.edu (C.R.); 2 Freelancer, 17404 Riells i Viabrea, Spain; jcollell23@gmail.com

**Keywords:** bullying, cyberbullying, primary education, perception of being a victim, victimization

## Abstract

This study aims to investigate victimization of bullying in primary school children, as well as its relationship with children’s perception of being a victim. In a sample of 4646 students from 3rd to 6th grade, we evaluated children’s victimization and cybervictimization behaviors, and children were also asked whether they had been victims of bullying or cyberbullying. From the participants, 36.7% were victims, and 4.4% cybervictims. In addition, 24.2% had a perception of being a victim, and 4.9% a perception of being a cybervictim. On the other hand, 56.9% of victims of traditional bullying had no perception of being a victim. The victimization behaviors of traditional bullying associated with a higher perception of being a victim were threats, while physical and direct verbal aggression implied a lower perception of being a victim. The results suggest the most frequent victimization behaviors may be normalized.

## 1. Introduction

Bullying is a type of aggressive behavior that implies intentionality, repetition and an imbalance of power between the aggressor and the victim, in a way that the victim is incapable of defending against the aggressor [1]. Cyberbullying occurs when bullying behaviors are carried out using electronic devices [2], situation in which the repetition, the imbalance of power and the roles of the participants are not as obvious as in traditional bullying [3]. For example, one aggression with electronic devices may be multiplied with only one intervention of the perpetrator [4]. Bullying may be divided into different types (physical, verbal and relational) each including direct and indirect forms of aggression, depending on whether the aggressor is concealed (indirect) or not (direct) [5,6]. In the present article we focus on the role of victims of bullying and cyberbullying, which implies notable negative consequences in their psychosocial adjustment [7]. To analyze this role we considered, on the one side, children’s perception of being a victim (the child admits to having been a victim or not), and on the other side, the victimization behaviors the child informs to have received. In this way, we intend to analyze victimization behaviors in primary school students as a function of sex and grade, as well as the relationship between victimization behaviors and the perception of having been a victim.

### 1.1. Experience of Bullying and Cyberbullying

An international report of UNESCO [8] informs of a global percentage of bullying of 32% at the age of 11 years. However, bullying prevalence varies as a function of the instruments used to measure it (which differ in crucial aspects such as the frequency criterion or the time reference period they use), the definition of the concept, the sex, school grade or country of the participants, among other variables [9,10,11]. Precisely, an extensive systematic review about cyberbullying in Spain concludes that it is of great importance to unify criteria and choose evaluation strategies appropriately [2]. For primary school, the study by Juvonen and Graham [12] suggests that between 9% and 25% of students are victims of school bullying, and these percentages could be even higher than in older children [13]. This represents an important health problem, as victims of bullying present depressive symptoms, suicidal ideation and suicide attempts (in some cases consummate), anxiety, school dropout and poor academic performance [14]. It has been reported that victims of bullying in primary school tend to experience more health problems (headache, dizziness, restlessness, nervousness and sleeping problems, among others) [15]. In relation to the different forms of bullying, as children grow old there is a change from direct bullying to indirect and relational bullying [6].

Regarding sex differences in traditional bullying, UNESCO [8] reports a higher prevalence of global victimization in boys than in girls. Specifically, it mentions the Global School-based Student Health Survey (GSHS) study, which finds a global prevalence of 34.8% in boys and 30.4% in girls (13 to 15 years old). The Health Behaviour in School-aged Children (HBSC) study for 2018 in Spain [16] also reports a higher a prevalence for boys (at the age of 11–12: 14.1% among girls and 17.1% among boys, and at the age of 13–14: 12.1% among girls and 15.7% among boys). Furthermore, a recent systematic review of studies about victimization in primary school found that in most of the studies boys are more likely to be victims of bullying than girls [14]. Sex differences may be due in part to the fact that boys tend to be involved in physical forms of aggression that are easier to identify, while girls tend to be involved in indirect forms of bullying in relational contexts [10]. On the other hand, research suggests that children in primary school have higher rates of victimization compared to older children [14].

In cyberbullying there are also important differences depending on the study [17], and UNESCO [8] reports a global cybervictimization of about 10%. In addition, the global report of the HBSC study in 2018 [18] found that girls were more likely than boys to be victims of cyberbullying (14% for girls and 12% for boys), especially at the age of 13. These sex differences were found in more than half of the countries/regions considered in the study. In Spain, the study by Romera et al. [19] reports a prevalence of cybervictimization in the last grades of primary school between 7.5% and 10%, and a higher cybervictimization in girls than in boys, both in secondary and primary school. Specifically for primary school, Navarro et al. [20,21,22] have found a prevalence of bullying cybervictimization between 4.6% and around 24%, differences that could be explained at least partially by methodological reasons such as the cut-off point for classifying a child as a victim.

Regarding the relationship between cyberbullying and age, the results of the literature seem quite inconsistent; while some studies have found an increase in cybervictimization as students grow old, others have found a decrease, but the majority reports a negligible association [23].

### 1.2. Perception of Being a Victim

Some students may receive different victimization behaviors with a certain frequency and still not consider themselves as being victims. Juvonen et al. [24] explain that those students who had a victim perception were evaluated worse in psychological well-being than those who did not consider themselves victimized. In another study [25], they found that victims were not conscious of the rejection they suffer from other children. In this sense, it is important that studies consider not only the victimization behaviors that children receive, but also whether children have the perception of being victimized, as it is useful for professionals and families to raise awareness and to alert about the danger of bullying behaviors [26]. Even so, only a few studies on harassment have taken into account the perception of the victims when identifying them. One of those is the study by Schuster [27], who found that, among 22 students (11–13 years old) who according to their classmates and teachers suffered bullying only 7 identified themselves as victims (32%). Another study, by Hwang et al. [28], found that in students aged 12 to 15 the percentage of victims (according to their classmates) who had a perception of being a victim was 16.2% (42 out of 259). Therefore, some studies have examined the perception of being a victim from the perspective of the victim compared to the view of the classmates, but we are unaware of studies exploring the perception of being a victim in relation to the victimization behaviors reported by the same child. This analysis could allow us to understand better how the different types of victimization behaviors are related to the perception of being a victim. On the other hand, Solberg and Olweus [29] argue that young children could have a more extensive conception of bullying than older children (including actions that are not intentional or not repeated), while other authors defend that younger students exclude indirect types of bullying [5,6]. In any case, the results of Solberg and Olweus suggest that boys and girls with low frequencies of bullying are less likely to consider that they are victims of bullying.

### 1.3. The Present Study

Although being bullied in primary school might have consequences in secondary school, and even adulthood [30], only a few studies on bullying and cyberbullying are focused on primary education, and they usually cover just the last cycle of this educational stage (5th and 6th grade). The present research also intends to provide data about bullying and cyberbullying in the middle cycle (3rd and 4th grade). Moreover, few studies have focused on the relationship between victimization behaviors and the perception of being a victim, which is important because the perception of being a victim plays an important role in the recognition of the bullying experience as well as in its consequences [28].

The present study has two main objectives: First, to study bullying and cyberbullying victimization behaviors in primary school students, and to analyze possible grade and sex differences, including the less studied 3rd and 4th grades; second, to examine the relationship between victimization behaviors and the students’ perception of being a victim.

In relation to the first objective, and according to the scientific literature reviewed above, we can formulate the following hypotheses for the sample of children from 3rd to 6th grade of primary school: (a) Boys will have higher victimization rates in traditional bullying compared to girls [14,16]; (b) Victimization rates in traditional bullying will decrease according to age [13,14]; (c) Girls will have higher cybervictimization rates compared to boys [18,19]; Finally, it was not possible to establish a clear hypothesis relating cyberbullying and age.

Regarding the second objective, we could not make a hypothesis, as we are not aware of prior research studying the relationship between self-reported victimization behaviors and having a perception of being a victim. Therefore, this part will be exploratory.

## 2. Materials and Methods

### 2.1. Participants

A total of 4646 students from 3rd to 6th grade of primary school in Catalonia, in northeastern Spain, participated in the study (49% girls). The age ranged between 7.33 and 13.42 years (M = 10.16; SD = 1.17). By grades, there were 1123 third-graders (49% girls; age: M = 8.62 years; SD = 0.37), 1167 fourth-graders (48.8% girls; age: M = 9.65 years; SD = 0.42), 1176 fifth-graders (49.1% girls; age: M = 10.59 years; SD = 0.40), and 1180 sixth-graders (49.4% girls; age: M = 11.61 years; SD = 0.44). Some participants did not respond the question about their sex (65 at 3rd, 55 at 4th, 56 at 5th and 59 at 6th), so results were calculated according to the available data.

With regard to the socioeconomic status, as we collected data on a representative sample of the students from 3rd to 6th grade of primary school in Catalonia, that took into account the type of school of the children as well as the school area (see Procedure section), we believe the sample is representative of the different socioeconomic statuses that exist in this area of Spain.

In order to ensure that our sample size was sufficient to detect an adequate effect, we computed a post hoc power analyses using G * Power 3.1 Software [31]. Results revealed that our total sample size of 4646 participants was sufficient, in the case of the ANOVA test, to detect with a 0.99 power a medium effect size (f = 0.25).

### 2.2. Instruments

It is worth noting that right before the administration of the questionnaires, a definition of bullying and cyberbullying was provided to the children by the researchers of the project. Children also had the opportunity to ask questions. Moreover, a definition was also written at the beginning of the questionnaires. The following instruments were administered to the participants, by order of presentation.

(a) Bullying Questionnaire

The European Bullying Intervention Project Questionnaire (EBIPQ) [32] was used. It is an instrument of 14 items (7 for victimization and 7 for aggression) in which children are asked what situations they have lived in the last two months, evaluated on a Likert scale with 5 response options: 0 = No; 1 = Yes, once or twice; 2 = Yes, once or twice a month; 3 = Yes, about once a week; 4 = Yes, more than once a week. In the present study, only the victimization scale has been taken into account. Reliability, assessed through Chronbach’s Alpha was 0.81 on the victimization scale.

(b) Cyberbullying Questionnaire

A reduced version of the European Cyberbullying Intervention Project Questionnaire (ECIPQ) [32] was used. The original instrument consists of 22 items (11 for cybervictimization and 11 for cyberaggression) evaluated on the same Likert scale mentioned for the bullying questionnaire. In our version for primary education (see Appendix A), some items were eliminated or merged, while others were maintained, finally using 12 items (6 cybervictimization and 6 cyberaggression). In this study, only cybervictimization items were taken into account. Reliability, assessed through Chronbach’s Alpha on the cybervictimization scale, was 0.75.

(c) Perception of being a victim and cybervictim

We asked children whether they have been a victim, aggressor or observer of bullying or cyberbullying. In relation to the perception of being a victim, we asked “Have you been a victim of bullying?” and “Have you been a victim of cyberbullying?” Children had to mark yes or no in each question.

### 2.3. Procedure

First, the Department of Education of the Catalan Government was contacted, who gave permission to conduct the study. To select the educational centers, a representative random sample of students from 3rd to 6th grade of primary school in Catalonia was carried out, stratified according to the type of school (public vs. private or concerted), and size (fewer than one class per grade, one class per grade, or more than one class per grade). Furthermore, in the case of public schools, the territorial area of the Department of Education was also considered as a criterion of representativeness. The school management teams were contacted to explain the project and request participation based on: (a) an explanatory letter of the study; (b) a letter of support from the Department of Education; and (c) consent from the school to confirm participation. A total of 41 schools participated in the study (data were collected between December 2018 and April 2019).

The questionnaires were administered mostly in paper, and only 10 group classes responded to it online. The families were informed of the objectives of the study and their informed consent was obtained. The researchers from the project administered the questionnaires in most cases, but five schools preferred to administer them on their own. After being provided with an explanation of bullying and cyberbullying, children chose to respond the questionnaires in Catalan or Spanish. Researchers from the project monitored that children answered correctly the questionnaires, and gave support to those who asked for help. At no time were the children asked for their names. After completing the questionnaires, children put them in a sealed envelope to preserve anonymity.

This study was approved by the Ethical and Biosecurity Research Committee of the University of Girona (code: CEBRU0016-2018).

### 2.4. Data Analysis

Data were analyzed using the SPSS 25 statistical program. To classify the participants into victims or cyber victims, the criteria by Romera et al. [19] was used, who considered as victims those participants with a minimum frequency of “Once or twice a month” in any of the EBIPQ or ECIPQ victimization items. On the other hand, in the line of García-Fernández et al. [33], participants with a minimum frequency of “More than once a week” in some item have also been considered as frequent victims or frequent cybervictims. Finally, the items of the EBIPQ questionnaire were classified into direct aggressive behaviors (items 1, 2 and 4) or indirect/relational behaviors (items 3, 5–7).

In order to compare victimization behaviors as a function of sex and grade, the mean scores of each type of behavior were used and an ANOVA of grade (4) × sex (2) was performed, also analyzing the simple effects of sex and grade. The partial eta squared (η_p_^2^) was taken as a measure of the effect.

To analyze the effects of grade and sex on the perception of the victim, the chi-square test was used, using Cramer’s V as a measure of the effect. The same test was used to analyze the relationship between victimization behaviors and perception of being a victim.

## 3. Results

### 3.1. Victims of Bullying and Cyberbullying

From the participants, 36.7% met the criteria established for victims of traditional bullying and 4.4% the criteria for cybervictims. On the other side, 17.4% of the participants were frequent victims of traditional bullying, while 1.7% of them were frequent cybervictims. Table 1 shows the victims of bullying and cyberbullying according to sex and grade. Table 2 shows the mean scores for victimization and cybervictimization items, so they represent for each sex and grade the mean frequency of the victimization and cybervictimization behaviors.

The results showed that 10.3% of victims of traditional bullying were also victims of cyberbullying. The prevalence of cyberbullying was much lower (of only 1%) among those participants who were not victims of traditional bullying. On the other hand, among the victims of cyberbullying, 85.8% were also victims of traditional bullying, while the prevalence of traditional bullying among participants who were not cybervictims was 34.1%.

The results in traditional bullying (means of victimization scores) showed a significant effect of sex (see Table 2), with boys reporting more frequent bullying, and a significant decrease according to grade (*F*(3, 4127) = 16.96; *p* < 0.001; η_p_^2^ = 0.012), there being no interaction between both variables (*p* = 0.445). The analysis of the simple effects of sex in each of the grades indicated no sex differences in 6th grade, but significant differences existed in the other grades. On the other hand, the analysis of the simple effects of the grade separated by sex presented a significant effect of grade both for boys (*F*(3, 4127) = 22.76; *p* <.001; η_p_^2^ = 0.016) and girls (*F*(3, 4127) = 38.71 *p* < 0.001; η_p_^2^ = 0.027), with a decrease in these behaviors in higher grades.

The results of the 2-way ANOVA for cyberbullying behaviors (mean scores) showed a significant effect of sex with higher scores for boys (see Table 2), a significant effect of grade (*F*(3, 4196) = 5.96; *p* < 0.001; η_p_^2^ = 0.004), and also an interaction between both variables (*F*(3, 4196) = 4.90, *p* = 0.002; η_p_^2^= 0.003). When analyzing the simple effects of sex in each of the grades, differences were observed between boys and girls at 3rd and 4th, but not at 5th or 6th grade (see Table 2). The analysis of the simple effects of grade for each sex indicated a grade effect for girls (*F*(3, 4196) = 8.38; *p* < 0.001, η_p_^2^ = 0.006) but not for boys (*p* = 0.063). Therefore, cybervictimization remained stable in boys throughout primary school, while increased in girls in the last years of this stage.

### 3.2. Victimization Behaviors

Table 3 shows the percentage of victims as a function of the number of victimization behaviors. From the total amount of victims of traditional bullying, more than half were victims in more than one item (60.5%). Similarly, 44.3% were frequent victims in more than one item. Most of the cybervictims and frequent cybervictims reported victimization behaviors just in one item (56.8% and 58.1% respectively).

Table 4 shows direct and indirect victimization behaviors. A two-way ANOVA of grade (4) × sex (2) was performed to analyze the effects of those variables. For direct victimization, there was an effect of sex and grade (*F*(3, 4309) = 34.55; *p* < 0. 001, η_p_^2^ = 0.023). The analysis of simple effects showed sex differences in each grade (see Table 2), and the analysis of the simple effects of the grade for boys and girls separately showed a decrease in direct victimization (as the grade increases) in both sexes (Girls: *F*(3, 4309) = 15.25, *p* < 0.001, η_p_^2^ = 0.011; Boys: *F*(3, 4309) = 20.77, *p* < 0.001, η_p_^2^ = 0.014). The interaction between the sex and grade variables was not statistically significant (*p* = 0.237). In summary, boys showed more direct victimization behaviors than girls, and in both sexes these behaviors tended to decrease from 3rd to 6th grade.

For indirect victimization (see Table 4), a sex effect was also found with higher scores for girls (see Table 4). Similarly, a grade effect was found (*F*(3, 4186) = 29.97, *p* < 0.001, η_p_^2^ = 0.021), but there was no interaction between sex and grade (*p* = 0.607). When analyzing the simple effects of grade for each sex, differences were found both in girls (*F*(1, 4186) = 13.38, *p* < 0.001, η_p_^2^ = 0.009) and boys (*F*(1, 4186) = 17.27, *p* < 0.001, η_p_^2^ = 0.012), with a decrease in both. Finally, the analysis of simple sex effects in each grade showed that sex differences in indirect victimization occurred only in 6th grade, with higher scores for girls. In Appendix B (Table A1) we show the descriptive statistics for each of the victimization and cybervictimization behaviors as a function of grade and sex. For the total sample, the most frequent victimization behaviors (of traditional bullying) were physical aggression (M = 0.77; SD = 1.10) and direct verbal aggression (M = 1.07; SD = 1.23).

### 3.3. Perception of Being a Victim

From all participants, 24.2% had the perception of having been a victim of traditional bullying, and 4.9% reported having been victims of cyberbullying. Table 5 shows the participants with a perception of being a victim as a function of grade and sex.

We did not find a relationship between sex and the perception of being a victim of traditional bullying (using the Chi-square test) in the total sample (*p* = 0.355), and only at 5th grade when dividing it by grades (3rd: *p* = 0.903; 4th: *p* = 0.536; 5th: χ^2^ (1, *n* = 1102) = 7.71, *p* = 0.006; Cramer’s V = 0.084; 6th: *p* = 0.818). However, there was a relationship between perception of being a victim and grade (χ^2^ (3, *n* = 4574) = 31.18, *p* < 0.001; Cramer’s V = 0.083). This relationship was significant both for boys (χ^2^ (3, *n* = 2209) = 19.75, *p* < 0.001; Cramer’s V = 0.095) and girls (χ^2^ (3, *n* = 2144) = 16.66, *p* = 0.001; Cramer’s V = 0.088), with a reduction in the perception of being a victims as the grade increased.

There was a relationship close to significant (χ^2^ (1, *n* = 4243) = 3.73, *p* = 0.053; Cramer’s V = 0.030) between the perception of being a cybervictim and sex (see Table 5). This relationship was significant at 3rd (χ^2^ (1, *n* = 993) = 8.83, *p* = 0.003; Cramer’s V = 0.094) and 4th grade (χ^2^ (1, *n* = 1063) = 5.71, *p* = 0.017; Cramer’s V = 0.073), with a higher perception of being a cybervictim in boys; it was not significant at 5th grade (*p* = 0.378), and it was significant again at 6th grade (χ^2^ (1, *n* = 1095) = 4.81, *p* = 0.028; Cramer’s V = 0.066), with a higher perception of being a cybervictim in girls. Besides, the perception of being a cybervictim was not significantly related to grade *(p* = 0.305), although when separating it by sex, grade differences in the perception of being a cybervictim were found both in boys and girls, with a reduction as the grade increases in boys (χ^2^ (3, *n* = 2149) = 8.93, *p* = 0.030; Cramer’s V = 0.064) and an increase in girls (χ^2^ (3, *n* = 2094) = 11.44, *p* = 0.010; Cramer’s V = 0.074).

### 3.4. Relation between Victimization and Perception of Being a Victim

When analyzing the relationship between the victimization behaviors reported in the EBIPQ questionnaire (on traditional bullying) and the perception of being a victim, a significant relationship was found between both variables (See Table 6), although the majority of victims (56.9%) according to the EBIPQ do not have the perception of having been a victim of bullying. This relationship remained stable throughout the different grades; the percentages of victims who did not have a perception of being a victim were 55.6% at 3rd, 61.5% at 4th, 54.5% at 5th, and 56.12% at 6th grade. Regarding the relationship between frequent victimization in traditional bullying and the perception of the victim, it was also statistically significant (See Table 6). In this case, frequent victims who did not have a perception of being a cybervictim were 44.6%, being quite stable throughout the grades (46.6% at 3rd, 51.7% at 4th, 43.3% at 5th, and 40.1% at 6th).

When analyzing the relationship between victimization and the perception of the victim as a function of the type of victimization behavior (see Table 7), we observed that the type of victimization behavior where the subjects had a greater perception of being a victim was being threatened, followed by exclusion/being ignored. On the other side, the behavior with the lowest perception of the victim was direct verbal aggression, and the second with the lowest perception was physical aggression, which are among the behaviors with a highest frequency (See Appendix Table A1).

The analysis of the relationship between the perception of being a victim of traditional bullying and the number of items in which participants had a suffered victimization (see Table 8) showed that the more items in which children reported victimization, the more likely they had a perception of being a victim.

### 3.5. Relation between Cybervictimization and Perception of Being a Cybervictim

Table 9 shows the relationship between cybervictims (according to the ECIPQ) and the perception of being a cybervictim. The Chi-square test shows a relationship between both variables. Even so, 67% of the cybervictims did not have the perception of being a cybervictim. Besides, a significant relationship was also found between being a frequent cybervictim and having the perception of being a cybervictim. The percentage of frequent cybervictims who did not have a perception of being a cybervictim was of 58.9%.

## 4. Discussion

This study aimed to analyze children’s victimization of bullying and cyberbullying in primary school (grades 3rd to 6th) as a function of grade and sex. Also, it intends to study the relationship between children’s victimization behaviors and their perception of being a victim.

### 4.1. Victimization in Traditional Bullying

We found 36.7% of victims of traditional bullying in children from 3rd to 6th grade (and 17.4% of frequent victims). These percentages are slightly higher than those indicated by UNESCO [8] globally at the age of 11 (32%), and also slightly higher than the report by Romera et al. [19] on 5th and 6th grade in Spain (around 30%). The traditional bullying victimization instruments and criteria in our study were equivalent to those of Romera et al., so the differences can possibly be explained by a greater victimization in our study in 3rd and 4th grades compared to 5th and 6th grades. Hence, our data point to a decrease in traditional bullying throughout primary school in both sexes, in line with other studies [8,11,14], and higher rates than in secondary school. Thus, our hypothesis about a decrease of victimization rates according to age has been supported. It is possible that bullying-related behaviors changes along primary school, but this change could also be explained by age-related changes in the conception of bullying [29].

Regarding sex differences, we found a higher victimization in traditional bullying in boys than in girls (except in 6th grade), similarly to other studies [8,14,16,34], placing the differences already from 3rd grade. Hence, although these data support our hypothesis of greater victimization in boys than in girls, this is not the case in 6th grade. Besides, the lack of sex differences in 6th grade could indicate a change in trend between primary and secondary education. In the study by Romera et al. [19], sex differences in traditional victimization were very low for primary education (girls showed 2% more victimization) but increased in secondary education (around 6–8% more for girls).

We found a greater direct victimization in boys than in girls, a difference that was significant in all grades. In contrast, girls showed more indirect victimization behaviors than boys, although these differences were only significant in 6th grade. These data are in line with some authors who suggest that boys are more involved in physical forms of aggression and girls in indirect forms [10]. Beyond these descriptive data, several questions emerge regarding the observed differences between boys and girls in the different grades and in relation to the different forms of bullying. Our study did not used the appropriate instruments to allow a discussion about this issue, but we consider that analyzing this phenomenon from a gender perspective would permit a better understanding of the issue [35].

### 4.2. Cybervictimization

We found 4.4% of prevalence of cybervictims (and 1.7% of frequent cybervictims), percentages lower than those found previously in other parts of Spain [21,22], and specifically in Andalusia, where the percentages in 5th and 6th of primary education varied from 7.5% to 10% [19]. On the other hand, while in our study the vast majority of cyberbullying victims were also victims of traditional bullying (85.8%), a very low percentage of victims of traditional bullying were also victims of cyberbullying (10.3%). Other studies already pointed out that in primary school cyberbullying is related to problems with traditional bullying [36], and in the present study we highlight the high probability of suffering traditional bullying among cybervictims. This might imply a lack of convenience of interventions only aimed at cybervictimization [37], especially in primary education.

With regard to sex differences in cybervictimization, our hypothesis has not been confirmed, as we did not observe more cybervictimization in girls compared to boys. Contrarily, we observed more cybervictimization in boys than in girls from 3rd and 4th grades. If we take into account both the effects of grade and sex, we found that cybervictimization remained stable in boys throughout primary education, while it increased from 4th grade in girls. So more cybervictimization was found for boys compared to girls in grades 3rd and 4th, but no sex differences existed in grades 5th or 6th, where an increase was found for girls, especially in grade 6. These results differ somewhat from other investigations in which a higher prevalence of cybervictimization was found for girls with older samples [8,16,19]. Thus, sex differences in cybervictimization could undergo a change in trend throughout primary and secondary education, going from being more frequent in boys first and in girls later. This suggests that teachers should be especially attentive to cybervictimization for boys in the earlier years of primary school, and according to what other studies suggest [38], it is important to implement prevention and intervention programs to help children understand the risks of communication technologies and to deal with cyberaggressions. Finally, in our study most cybervictims were victims of only one type of behavior, whereas most victims of traditional bullying were victims of more than one behavior.

### 4.3. Relation between Victimization and Perception of Being a Victim in Traditional Bullying

In our study we found that the perception of being a victim (of traditional bullying) is reduced as the grade increases, in line with previous results [39,40]. Considering that the question about perception of being a victim refers to any time point, this reduction is counterintuitive (because older participants have had a longer period for having suffered bullying), and might indicate that younger children have a broader conception of bullying than older children [41]. Nevertheless, it could also be interpreted as a greater reluctance in older children to accept that they have been a victim. In this sense, some school bullying prevention programs include public recognition of the prevalence of bullying, so that the victim does not attribute it so much to oneself and more to contextual conditions [24]. It is also possible that in the case of the youngest children (3rd grade), a different conception or understanding of bullying may have influenced the results.

Our results show that only 43.1% of victims of traditional bullying (according to children’s informed victimization behaviors) have a perception of being a victim (and 55.4% in the case of frequent victims). These percentages are higher than in the studies by Schuster [27] and Hwang et al. [28], so a greater congruence between the experience of victimization and the perception of being a victim appears when the victimization is evaluated through self-report (as in our case) than through peers (as in the other mentioned studies).

Our study highlights that the perception of being a victim is related to the type of victimization behaviors. Thus, the most frequent victimization behaviors (physical aggression and direct verbal aggression) had a lower perception of being a victim, perhaps indicating a normalization of those behaviors. In this direction, Cuadrado [42] states that many children and adolescents internalize and normalize many indirect verbal and physical abuses. Perhaps this could also explain why more than half of the victims in our study did not have a perception of being a victim, and only when the number of victimization behaviors increased, or if they suffered a more frequent victimization, this perception increased. Solberg and Olweus [29] also found that boys and girls with low frequencies of bullying were less likely to consider themselves as victims.

### 4.4. Relationship between Cybervictimization and Perception of Being a Cybervictim

The perception of being a cybervictim was higher in boys compared to girls at 3rd and 4th grades, but higher in girls at 6th grade, following a trend to decrease in boys throughout primary school and to increase in girls. These data are similar to our results on cybervictimization prevalence. Besides, we observed that only 33% of cybervictims (and 41.1% of frequent cybervictims) had a perception of being a cybervictim, which is lower than in perception of being a victim in traditional bullying. This is especially worrisome considering that attacks with electronic devices can multiply and lengthen over time with a single action by the aggressor [4]. Thus, it seems necessary to work in primary schools on the concept of being a victim of bullying, and especially of cyberbullying.

### 4.5. Limitations

Regarding the limitations of the study, when we asked about the perception of being a victim of bullying, we did not ask how long ago it happened, which could have helped to interpret our results more adequately. In addition, the instruments used to measure victimization (EBIQP) and cybervictimization (ECIPQ) do not directly address the issues of intentionality and imbalance of power inherent in the definition of bullying. Moreover, if we had used sociometric data, or measured the victimization perceived by the peers, our data would have been more comprehensive. Finally, as we commented earlier, we cannot discuss up to which point the observed sex differences are related to gender socialization—see Navarro [43] for a review of gender studies on cyberbullying.

## 5. Conclusions

In the present study we found a reduction with grade of both children’s victimization behaviors (of traditional bullying) and of children’s perception of (traditional) bullying. Future research should try to disentangle whether these age-related changes are an effect of behavioral or conceptual changes. It is possible that young children have a broader concept of bullying compared to older children, but also that older children are more reluctant to accept that they have been bullied. Regarding sex differences in traditional bullying, boys were more victimized than girls except in 6th grade. Also, in line with prior research, boys suffered more direct victimization (physical and verbal aggressions) than girls in all grades, while girls in 6th grade suffered more indirect victimization than boys. In any case, direct victimization was more frequent than indirect victimization in both sexes.

Cybervictimization was stable in boys but increased in girls along the primary school years. As it was more frequent in boys only at 3rd and 4th grade, and prior studies had found a higher prevalence for girls in older samples [8,19], cybervictimization could undergo a sex-related change of tendency from primary to secondary education. On the other hand, our results highlight that cybervictims in primary school have a high likelihood of suffering from traditional bullying, an aspect that should be considered in interventions designed to address cyberbullying.

We also found that the victimization behaviors of traditional bullying that were associated with a higher perception of being a victim were threats; on the other hand, physical and direct verbal aggression, which were among the behaviors with the highest frequency, were associated with a lower perception of being a victim. This might indicate a normalization of these aggressive behaviors in primary school children, which is an important finding. According to Thornberg [44], children often view bullying as a normal consequence of deviance related to the victim or fight for social status, and thus they might interpret it as ordinary and trivial and reduce their motives for intervention. Future studies could analyze further which factors contribute to the normalization of these behaviors, and also to understand its consequences on children. More effort must be made to understand how children perceive victimization so that education professionals and families can reduce the widespread tolerance and consequent normalization of bullying [26].

From the Theory of system justification [45] it is argued that it is painful to accept that one is living an injustice situation, which leads some people to interpret this situation less negatively; this might help them to feel better, but at the same time reduces the likelihood of taking action and rejecting injustice, especially if they view the situation as inevitable. This could (at least in part) explain why we found that only about half of frequent victims/cybervictims have the perception of having been a victim/cybervictim. Thornberg [44] suggests that bullying might create a *victim career* on the victim, which starts with a social construction of being deviant or marginalized, and which implies a social devaluation of the victim. In this regard, our results suggest the need for psychoeducational interventions to work on children’s conceptions of being a victim of bullying (e.g., to help valuing themselves) and against the acceptance and normalization of aggressive behaviors. This seems especially necessary in virtual contexts, where we found a lower perception of being a victim. School bullying represents an important health problem for the victims [14,15]. In this regard, educational centers should promote the rejection of these behaviors and offer explicit support for those who suffer victimization behaviors, as it would make it easier for them to seek help [46].

## Figures and Tables

**Table 1:** Percentages of victims and cybervictims as a function of grade and sex.

Grade	Sex	Victims	Cybervictims	Frequent Victims	Frequent Cybervictims
3rd	Girls	41%	3%	21%	1%
		*n* = 212	*n* = 16	*n* = 109	*n* = 7
	Boys	53%	7%	29%	2%
		*n* = 286	*n* = 34	*n* = 158	*n* = 11
4th	Girls	31%	2%	12%	1%
		*n* = 167	*n* = 10	*n* = 69	*n* = 3
	Boys	42%	5%	17%	2%
		*n* = 237	*n* = 25	*n* = 95	*n* = 9
5th	Girls	27%	3%	13%	1%
		*n* = 147	*n* = 16	*n* = 70	*n* = 7
	Boys	42%	5%	19%	2%
		*n* = 239	*n* = 29	*n* = 110	*n* = 11
6th	Girls	26%	6%	12%	3%
		*n* = 144	*n* = 32	*n* = 67	*n* = 14
	Boys	33%	5%	14%	2%
		*n* = 187	*n* = 28	*n* = 82	*n* = 10

Percentages represent number of victims or cybervictims for each sex within each grade.

**Table 2:** Means (and SD) of bullying and cyberbullying victimization frequency as a function of grade and sex.

Grade	Sex	Victimization		Cybervictimization	
		Means (SD)	ANOVA Comparison by Sex	Means (SD)	ANOVA Comparison by Sex
3rd	Girls	0.75 (0.77)	*F*(1, 4127) = 5.39 *p* = 0.020; η_p_^2^ = 0.001	0.06 (0.23)	F (1, 4196) = 13.29, *p* < 0.001; η_p_^2^ = 0.003
	Boys	0.84 (0.73)		0.12 (0.40)	
4th	Girls	0.54 (0.62)	*F*(1, 4127) = 5.20 *p* = 0.023, η_p_^2^ = 0.001	0.04 (0.15)	*F*(1, 4196) = 4.57 *p* = 0.033; η_p_^2^ = 0.001
	Boys	0.64 (0.61)		0.08 (0.21)	
5th	Girls	0.49 (0.59)	*F*(1, 4127) = 8.68; *p* = 0.003; η_p_^2^ = 0.002	0.07 (0.22)	*p* = 0.084
	Boys	0.61 (0.67)		0.10 (0.28)	
6th	Girls	0.49 (0.63)	*p* = 0.450	0.12 (0.30)	*p* = 0.124
	Boys	0.52 (0.62)		0.10 (0.28)	
Total	Girls	0.56 (0.65)	*F*(1, 4127) = 17.31 *p* < 0.001; η_p_^2^ = 0.004	0.07 (0.23)	*F*(1, 4196) = 9.34 *p* = 0.002; η_p_^2^ = 0.002
	Boys	0.65 (0.67)		0.10 (0.30)	

Descriptive data represent the mean scores of the frequencies in all victimization/cybervictimization items (range: 0 to 4).

**Table 3:** Percentages of victims and frequent victims as a function of the number of victimization behaviors.

Number of Victimization Behaviors	Victims	Frequent Victims	Cybervictims	Frequent Cybervictims
1	39.5%	55.7%	56.8%	58.1%
2	24.1%	26%	23.4%	23%
3	15.7%	8%	12.5%	12.2%
4	10.2%	5.3%	3.6%	4.1%
5	5.6%	3.1%	2.6%	1.4%
6	3%	1.7%	1%	1.4%
7	1.6%	0.1%		

Percentages were calculated according to the total: (a) victims of traditional bullying; (b) frequent victims of traditional bullying; (c) cybervictims; and (d) frequent cybervictims.

**Table 4:** Direct and indirect victimization behaviors in traditional bullying according to grade and sex.

Grade	Sex	Direct Victimization		Indirect Victimization	
		Means (SD)	ANOVA Comparison by Sex	Means (SD)	ANOVA Comparison by Sex
3rd	Girls	0.83 (0.92)	*F*(1, 4309) = 25.92 *p* < 0.001, η_p_^2^ = 0.006	0.69 (0.77)	*p* = 0.489
	Boys	1.1 (0.99)		0.66 (0.69)	
4th	Girls	0.58 (0.74)	*F*(1, 4309) = 26.94 *p* < 0.001, η_p_^2^ = 0.010	0.52 (0.63)	*p* = 0.438
	Boys	0.85 (0.84)		0.49 (0.61)	
5th	Girls	0.55 (71)	*F*(1, 4309) = 43.78 *p* < 0.001, η_p_^2^ = 0.010	0.45 (0.61)	*p* = 0.682
	Boys	0.88 (0.93)		0.43 (0.63)	
6th	Girls	0.52 (0.71)	*F*(1, 4309) = 13.80 *p* < 0.001, η_p_^2^ = 0.003	0.48 (0.63)	*F*(1, 4186) = 4.73 *p* = 0.030, η_p_^2^ = 0.001
	Boys	0.70 (0.83)		0.39 (0.58)	
Total	Girls	0.62 (0.78)	*F*(1, 4309) = 106.22 *p* < 0.001, η_p_^2^ = 0.024	0.53 (0.67)	*F*(1, 4186) = 4.07 *p* = 0.044, η_p_^2^ = 0.001
	Boys	0.88 (0.91)		0.49 (0.64)	

Scores ranged from 0 to 4.

**Table 5:** Percentages and frequencies of participants with a perception of being a victim or cybervictim according to grade and sex.

Grade	Sex	Perception of Victim	Perception of Cybervictim
		Percentages and Frequencies	Percentages and Frequencies
3rd	Girls	30% *n* = 155	3% *n* = 17
	Boys	29% *n* = 158	7% *n* = 39
4th	Girls	21% *n* = 113	2% *n* = 13
	Boys	19% *n* = 109	5% *n* = 29
5th	Girls	21% *n* = 113	5% *n* = 25
	Boys	27% *n* = 156	6% *n* = 32
6th	Girls	23% *n* = 128	6% *n* = 35
	Boys	23% *n* = 128	4% *n* = 20

Percentages represent number of children with a perception of being a victim or cybervictim for each sex.

**Table 6:** Relationship between victimization and perception of being a victim.

Type of victim		Perception of Victim		Chi-Square
		No	Yes	
Victim	Yes	954	722	χ^2^ (1, *n* = 4574) = 515.33 *p* < 0.001; Cramer’s V = 0.336
	No	2514	384	
Frequent victim	Yes	342	424	χ^2^ (1, *n* = 4574) = 487.67 *p* < 0.001; Cramer’s V = 0.327).
	No	3126	682	

**Table 7:** Percentages of victims who have a perception of being a victim as a function of the type of behavior.

Type of Victimization	Perception of Being a Victim	
	No	Yes
Physical aggression *n* = 129	71.3%	28.7%
Direct verbal aggression *n* = 249	75.5%	24.5%
Indirect verbal aggression *n* = 49	63.3%	36.7%
Being threatened *n* = 24	45.8%	54.2%
Being robbed or having objects broken *n* = 37	67.6%	32.4%
Being excluded or ignored *n* = 81	59.3%	40.7%
Spreading rumors *n* = 39	69.2%	30.8%

**Table 8:** Percentage of victims with a perception of being a victim according to the number of victimization behaviors.

Number of Victimization Behaviors	Victims Who Have a Perception of Being a Victim	Frequent Victims Who Have a Perception of Being a Victim
1 item	30.6	49.2
2 items	40.6	58.7
3 items	47.5	75.4
4 items	58.3	62.2
5 items	67.8	71.4
6 items	72.3	83.3
7 items	91.7	100

**Table 9:** Relationship between victimization and perception of being a cybervictim.

Type of Victim		Perception of Being a Cybervictim		Chi-Square
		No	Yes	
Victim	Yes	132	65	χ2 (1, *n* = 4333) = 352.42, *p* < 0.001; Cramer’s V = 0.285
	No	3990	146	
Frequent Victim	Yes	43	30	χ2 (1, *n* = 4314) = 214.11 *p* < 0.001; Cramer’s V = 0.223
	No	4064	177	

## Appendix A

Items used in the cyberbullying questionnaire.

1. Someone has said offensive words, insulted or threatened me using social networks or WhatsApp.
2. Someone has spoken badly about me using social networks or WhatsApp.
3. Someone has tried to impersonate me on social media or WhatsApp.
4. Someone has posted personal information about me on the Internet without my permission.
5. Someone has posted compromising videos or photos of me on the Internet.
6. I have been excluded or ignored from a social network or chat.
7. I have said offensive words to someone, I have insulted or threatened them using social networks or WhatsApp.
8. I have spoken badly of someone using social networks or WhatsApp.
9. I have tried to impersonate someone on social networks or WhatsApp to harm another person.
10. I have posted someone’s personal information on the Internet without their permission.
11. I have posted compromised videos or photos of someone on the Internet.
12. I have excluded or ignored someone on a social network or chat.

## Appendix B

**Table A1:** Mean (and SD) of the different victimization and cybervictimization behaviors as a function of grade and sex.

	3rd			4th			5th			6th		
	Girls	Boys	Total	Girls	Boys	Total	Girls	Boys	Total	Girls	Boys	Total
**Traditional bullying**												
Physical aggression	0.93 (1.24)	1.30 (1.36)	1.12 (1.323)	0.60 (0.96)	0.82 (1.08)	0.72 (1.04)	0.54 (0.89)	0.87 (1.11)	0.70 (1.01)	0.43 (0.86)	0.64 (0.95)	0.54 (0.91)
Direct verbal aggression	0.96 (1.22)	1.34 (1.36)	1.16 (1.31)	0.80 (1.08)	1.20 (1.24)	1.00 (1.18)	0.89 (1.10)	1.3 (1.33)	1.08 (1.23)	0.87 (1.14)	1.13 (1.26)	1.02 (1.22)
Indirect verbal aggression	0.61 (1.03)	0.61 (1.02)	0.61 (1.03)	0.53 (0.95)	0.61 (1.03)	0.58 (1.01)	0.54 (0.94)	0.55 (1.02)	0.55 (0.98)	0.59 (0.95)	0.49 (0.92)	0.55 (0.94)
Being threatened	0.62 (0.99)	0.68 (1.04)	0.64 (1.01)	0.34 (0.75)	0.52 (0.89)	0.43 (0.83)	0.25 (0.64)	0.50 (0.94)	0.37 (0.82)	0.24 (0.63)	0.34 (0.76)	0.29 (0.70)
Robbery or breaking objects	0.57 (0.96)	0.63 (0.98)	0.59 (0.97)	0.35 (0.69)	0.37 (0.72)	0.37 (0.72)	0.29 (0.66)	0.25 (0.63)	0.27 (0.65)	0.19 (0.49)	0.23 (0.56)	0.21 (0.54)
Being excluded or ignored	0.88 (1.11)	0.74 (1.05)	0.80 (1.07)	0.67 (0.98)	0.54 (0.91)	0.60 (0.94)	0.58 (0.92)	0.55 (0.98)	0.55 (0.94)	0.61 (1.00)	0.43 (0.86)	0.52 (0.94)
Spreading rumors	0.74 (1.06)	0.73 (1.07)	0.73 (1.07)	0.53 (0.92)	0.45 (0.80)	0.49 (0.86)	0.44 (0.84)	0.45 (0.85)	0.44 (0.85)	0.56 (0.90)	0.46 (0.88)	0.51 (0.89)
**Cyberbullying**												
Direct verbal aggression	0.08 (0.42)	0.14 (0.57)	0.12 (0.51)	0.05 (0.32)	0.11 (0.40)	0.08 (0.36)	0.11 (0.45)	0.19 (0.63)	0.14 (0.54)	0.16 (0.51)	0.16 (0.58)	0.16 (0.55)
Indirect verbal aggression	0.07 (0.40)	0.13 (0.54)	0.11 (0.48)	0.06 (0.27)	0.07 (0.32)	0.06 (0.30)	0.09 (0.39)	0.13 (0.53)	0.11 (0.46)	0.23 (0.66)	0.13 (0.48)	0.18 (0.57)
Impersonation	0.05 (0.35)	0.12 (0.50)	0.09 (0.43)	0.04 (0.28)	0.05 (0.29)	0.05 (0.30)	0.05 (0.34)	0.05 (0.28)	0.05 (0.31)	0.06 (0.36)	0.06 (0.34)	0.06 (0.37)
Posting personal information	0.06 (0.33)	0.12 (0.50)	0.09 (0.43)	0.05 (0.27)	0.04 (0.27)	0.04 (0.27)	0.03 (0.22)	0.05 (0.33)	0.04 (0.28)	0.05 (0.30)	0.06 (0.33)	0.05 (0.31)
Posting photos or videos	0.08 (0.43)	0.10 (0.42)	0.09 (0.44)	0.02 (0.17)	0.07 (0.33)	0.04 (0.26)	0.02 (0.15)	0.06 (0.33)	0.04 (0.26)	0.04 (0.25)	0.05 (0.30)	0.15 (0.30)
Being excluded or ignored	0.06 (0.36)	0.15 (0.55)	0.11 (0.46)	0.06 (0.261)	0.14 (0.52)	0.10 (0.42)	0.12 (0.448)	0.14 (0.47)	0.13 (0.47)	0.18 (0.55)	0.13 (0.43)	0.16 (0.49)

## References

[1] Smith P.K., Brain P. (2000). Bullying in schools: Lessons from two decades of research. Aggress Behav..

[2] Zych I., Ortega-Ruiz R., Marín-López I. (2016). Cyberbullying: A systematic review of research, its prevalence and assessment issues in Spanish studies. Psicol. Educ..

[3] Slonje R., Smith P.K., Frisén A. (2013). The nature of cyberbullying, and strategies for prevention. Comput. Hum. Behav..

[4] Menesini E., Nocentini A., Palladino B.E., Frisén A., Berne S., Ortega-Ruiz R., Calmaestra J., Scheithauer H., Schultze-Krumbholz A., Luik P. (2012). Cyberbullying definition among adolescents: A comparison across six European countries. Cyberpsychol. Behav. Soc. Netw..

[5] Björkqvist K., Lagerspetz K.M., Kaukiainen A. (1992). Do girls manipulate and boys fight? Developmental trends in regard to direct and indirect aggression. Aggress Behav..

[6] Rivers I., Smith P.K. (1994). Types of bullying behavior and their correlates. Aggress Behav..

[7] Cook C.R., Williams K.R., Guerra N.G., Kim T.E., Sadek S. (2010). Predictors of bullying and victimization in childhood and adolescence: A meta-analytic investigation. Sch. Psychol. Q..

[8] UNESCO (2019). Behind the Numbers: Ending School Violence and Bullying.

[9] Hymel S., Swearer S.M. (2015). Four decades of research on school bullying: An introduction. Am. Psychol..

[10] Menesini E., Salmivalli C. (2017). Bullying in schools: The state of knowledge and effective interventions. Psychol. Health Med..

[11] Smokowski P.R., Evans C.B.R. (2019). Bullying and Victimization across Lifespan. Playground Politics and Power.

[12] Juvonen J., Graham S. (2014). Bullying in schools: The power of bullies and the plight of victims. Annu. Rev. Psychol..

[13] Robers S., Kemp J., Truman J. (2013). Indicators of School Crime and Safety: 2012 (NCES 2013-036/NCJ 241446).

[14] Suárez-García Z., Álvarez-García D., Rodríguez C., de Lleida U. (2020). Predictores de ser víctima de acoso escolar en Educación Primaria: Una revisión sistemática. Rev. Psicol. Educ. Online Adv..

[15] Karatas H., Ozturk C. (2011). Relationship Between Bullying and Health Problems in Primary School Children. Asian Nurs. Res..

[16] Moreno C., Ramos P., Rivera F., Sánchez-Queija I., Jiménez-Iglesias A., García-Moya I., Moreno-Maldonado C., Paniagua C., Villafuerte-Díaz A., Ciria-Barreiro E. (2019). Informe Comparativo de las Ediciones 2002-2006-2010-2014-2018 del Estudio HBSC en España.

[17] Brochado S., Soares S., Fraga S. (2017). A scoping review on studies of cyberbullying prevalence among adolescents. Trauma Violence Abuse.

[18] Inchley J., Currie D., Budisavljevic S., Torsheim T., Jåstad A., Cosma A. (2020). Spotlight on adoLescent Health and Well-Being. Findings from the 2017/2018 Health Behaviour in School-Aged Children (HBSC) Survey in Europe and Canada.

[19] Ruiz R.O., del Rey Alamillo R., Bolaños J.A.C., Almanzor C.V., Ortiz O.G., Alcaide F.C., Zych I., Fernández C.M.G., González R.L. (2017). Bullying, Cyberbullying y Dating Violence. Estudio de la Gestión de la vida Social Estudiantes de Primaria y Secundaria en Andalucía.

[20] Navarro R., Ruiz-Oliva R., Larrañaga E., Yubero S. (2015). The impact of cyberbullying and social bullying on optimism, global and school-related happiness and life satisfaction among 10–12-year-old. Appl. Res. Q. Life.

[21] Navarro R., Serna C., Martínez V., Ruiz-Oliva R. (2013). The role of Internet use and parental mediation on cyberbullying victimization among Spanish children from rural public schools. Eur. J. Psychol. Educ..

[22] Navarro R., Yubero S., Larrañaga E., Martínez V. (2012). Children’s cyberbullying victimization: Associations with social anxiety and social competence in a Spanish sample. Child. Indic Res..

[23] Payne A., Hutzell K. (2015). Old Wine, New Bottle? Comparing Interpersonal Bullying and Cyberbullying Victimization. Youth Soc..

[24] Juvonen J., Nishina A., Graham S., Juvonen J., Graham S. (2001). Self-views versus peer perceptions of victim status among early adolescents. Peer Harassment in School: The Plight of the Vulnerable and Victimized.

[25] Ros M., Jiménez T., González A., Rodríguez J. (2017). Relación entre el bullying y el estado emocional y social en niños de educación primaria. Revista de Psicología Clínica con Niños y Adolescentes.

[26] Khan R., Rogers P. (2015). The Normalization of Sibling Violence: Does Gender and Personal Experience of Violence Influence Perceptions of Physical Assault Against Sibling. J. Interpers. Violence.

[27] Schuster B. (1999). Outsiders at school: The prevalence of bullying and its relation with social status. Group Process. Intergroup Relat..

[28] Hwang S., Kim Y.S., Koh Y.J., Bishop S., Leventhal B.L. (2017). Discrepancy in perception of bullying experiences and later internalizing and externalizing behavior: A prospective study. Aggress Behav..

[29] Solberg M.E., Olweus D. (2003). Prevalence estimation of school bullying with the Olweus Bully/Victim Questionnaire. Aggress Behav..

[30] Healy K.L., Grzazek O.Y., Sanders M.R. (2020). Attributions for improvement in children bullied at school. J. Sch. Violence.

[31] Faul F., Erdfelder E., Lang A., Buchner A. (2007). G*Power 3: A flexible statistical power analysis program for the social, behavioral, and biomedical sciences. Behav. Res. Methods.

[32] Ortega-Ruiz R., Del Rey R., Casas J.A. (2016). Evaluar el bullying y el cyberbullying validación española del EBIP-Q y del ECIP-Q. Psicol. Educ..

[33] García-Fernández C.M., Romera Félix E.M., Ortega-Ruiz R. (2015). Explicative factors of face-to-face harassment and cyberbullying in a sample of primary students. Psicothema.

[34] Smith P.K., López-Castro L., Robinson S., Görzig A. (2019). Consistency of gender differences in bullying in 480 cross-cultural surveys. Aggress Violent Behav..

[35] Mishna F., Schwan K.J., Birze A., Van Wert M., Lacombe-Duncan A., McInroy L., Attar-Schwartz S. (2020). Gendered and Sexualized Bullying and Cyber Bullying: Spotlighting Girls and Making Boys Invisible. Youth Soc..

[36] García-Fernández C.M., Romera Félix E.M., Ortega-Ruiz R. (2016). Relaciones entre el bullying y el cyberbullying: Prevalencia y co-ocurrencia. Pensam. Psicológico.

[37] Modecki K.L., Minchin J., Harbaugh A.G., Guerra N.G., Runions K.C. (2014). Bullying prevalence across contexts: A meta-analysis measuring cyber and traditional bullying. J. Adolesc. Health.

[38] Machimbarrena J., Garaigordobil M. (2018). Prevalence of Bullying and Cyberbullying in the Last Stage of Primary Education in the Basque Country. Span. J. Psychol..

[39] Analitis F., Velderman M.K., Ravens-Sieberer U., Detmar S., Erhart M., Herdman M., Berra S., Alonso J., Rajmil L., The European Kidscreen Group (2009). Being Bullied: Associated Factors in Children and Adolescents 8 to 18 Years Old in 11 European Countries. Pediatrics.

[40] Pervanidou P., Makris G., Bouzios I., Chrousos G., Roma E., Chouliaras G. (2019). Bullying victimization: Associated contextual factors in a Greek sample of children and adolescents. Psychiatriki.

[41] Smith P.K., Levan S. (1995). Perceptions and experiences of bullying in younger pupils. Br. J. Educ. Psychol..

[42] Cuadrado I. (2011). Divergence in aggressors’ and victims’ perceptions of bullying: A decisive factor for differential psychosocial intervention. Child. Youth Serv. Rev..

[43] Navarro R., Navarro R., Yubero S., Larrañaga E. (2016). Gender Issues and Cyberbullying in Children and Adolescents: From Gender Differences to Gender Identity Measures. Cyberbullying across the Globe.

[44] Thornberg R. (2010). Schoolchildren’s social representations on bullying causes. Psychol. Sch..

[45] Jost J., Sapolsky R., Nam H. (2018). Speculations on the evolutionary origins of system justification. Evol. Psychol..

[46] Unnever J.D., Cornell D.G. (2004). Middle school victims of bullying: Who reports being bullied?. Aggress Behav..

